# Reviewer acknowledgement 2015

**DOI:** 10.1186/s40337-016-0092-4

**Published:** 2016-02-19

**Authors:** Phillipa Hay, Stephen Touyz

**Affiliations:** School of Medicine, Centre for Health Research, Western Sydney University, Sydney, Australia; School of Medicine, James Cook University, Townsville, Australia; Schools of Psychology and Centre for Eating and Dieting Disorders (Boden Institute), University of Sydney, Sydney, Australia

## Abstract

The *Journal of Eating Disorders* editorial team would like to thank all reviewers who have contributed to the journal in 2015.

**Samir Al-Adawi**

Oman

**Karina Allen**

Australia

**Jon Arcelus**

United Kingdom

**Evelyn Attia**

United States

**Rachel Bachner-Melman**

Israel

**Roni Barak**

Israel

**Carolyn Becker**

United States

**Carl Birmingham**

Canada

**Chris Boyatzis**

United States

**Caroline Braet**

Belgium

**Juleen Buser**

United States

**Susan Byrne**

Lebanon

**Iain Campbell**

United Kingdom

**Terry Carney**

Australia

**Jue Chen**

China

**Simon Clarke**

Australia

**Angelica Claudino**

Brazil

**Loa Clausen**

Denmark

**Jennifer Coelho**

Canada

**Myra Cooper**

United Kingdom

**Ross Crosby**

United States

**Riccardo Dalle Grave**

Italy

**Martina de Zwaan**

Germany

**Linley Denson**

Australia

**Ivan Eisler**

United Kingdom

**Manfred Fichter**

Germany

**Sarah Fogarty**

Australia

**David Forbes**

Australia

**Katrin Giel**

Germany

**Scott Griffiths**

Australia

**Kjersti Solhaug Gulliksen**

Norway

**Phillipa Hay**

Australia

**Anja Hilbert**

Germany

**Vincent Ho**

Australia

**Hans Hoek**

Netherlands

**Frank Hume**

Australia

**Carokine Hunt**

Australia

**Mimi Israel**

Canada

**David Jimerson**

United States

**Allan Kaplan**

Canada

**Michael Kohn**

Australia

**Hubert Lacey**

United Kingdom

**Janet Latner**

United States

**Yael Latzer**

Israel

**Anna Lavis**

United Kingdom

**Daniel Le Grange**

United States

**Tanja Legenbauer**

Germany

**Sau Fong Leung**

Hong Kong

**Paolo Machado**

Portugal

**Sloane Madden**

Australia

**Sarah Maguire**

Australia

**Sian McLean**

Australia

**Philip Mehler**

United States

**Jennifer Mills**

Canada

**Deborah Mitchison**

Australia

**Adith Mohan**

Australia

**Stuart Murray**

Australia

**Richard Newton**

Australia

**Marion Olmsted**

Canada

**Susan Paxton**

Australia

**Kevin Pile**

Australia

**Carolyn Plateau**

United Kingdom

**Cecile Rausch Herscovici**

Argentina

**Deborah Reas**

Norway

**Gaby Resmark**

Germany

**Paul Rhodes**

Australia

**Lina Ricciardelli**

Australia

**P Scott Richards**

United States

**Elizabeth Rieger**

Australia

**Melissa Rouel**

Australia

**Pratap Sharan**

India

**Finns Skarderud**

Norway

**Evelyn Smith**

Australia

**Nerissa Soh**

Australia

**Kristin Stedal**

Norway

**Daniel Stein**

United States

**Esben Strodl**

Australia

**Lois Surgenor**

New Zealand

**Amy Talbot**

Australia

**Martin Teufel**

Germany

**Michael Theodoros**

Australia

**Tracey Wade**

Australia

**Glenn Waller**

United Kingdom

**Yan-Fang Wang**

China

**Hunna Watson**

Australia

**Martin Weltman**

Australia

**Steve Wonderlich**

United States

**Blake Woodside**

Canada

**Sarah Young**

Australia

**Florian Zepf**

Australia

**Stephan Zipfel**

Germany

